# Muscle metabolome and adipose tissue mRNA expression of lipid metabolism-related genes in over-conditioned dairy cows differing in serum-metabotype

**DOI:** 10.1038/s41598-021-90577-w

**Published:** 2021-05-27

**Authors:** Hassan Sadri, Morteza Hosseini Ghaffari, Katharina Schuh, Christian Koch, Helga Sauerwein

**Affiliations:** 1 Department of Clinical Science, Faculty of Veterinary Medicine, University of Tabriz, 516616471 Tabriz, Iran; 2 Institute of Animal Science, Physiology Unit, University of Bonn, 53115 Bonn, Germany; 3 Department of Life Sciences and Engineering, Animal Nutrition and Hygiene Unit, University of Applied Sciences Bingen, 55411 Bingen am Rhein, Germany; 4 Educational and Research Centre for Animal Husbandry, Hofgut Neumuehle, 67728 Muenchweiler an der Alsenz, Germany

**Keywords:** Fat metabolism, Metabolomics

## Abstract

Over-conditioned dairy cows, classified by body condition score (BCS) and backfat thickness (BFT) are less able to metabolically adapt to the rapidly increasing milk yield after parturition. Based on serum metabolome and cluster analyses, high BCS cows (HBCS) could be classified into metabotypes that are more similar to normal (NBCS) cows, i.e., HBCS predicted normal (HBCS-PN) than the HBCS predicted high (HBCS-PH) cows—similar to the concept of obese but metabolically healthy humans. Our objective was to compare muscle metabolome and mRNA abundance of genes related to lipogenesis and lipolysis in adipose tissue between HBCS-PH (n = 13), HBCS-PN (n = 6), and NBCS-PN (n = 15). Tail-head subcutaneous fat was biopsied on d −49, 3, 21, and 84 relative to parturition. Potential differences in the oxidative capacity of skeletal muscle were assessed by targeted metabolomics in *M. semitendinosus* from d 21. Besides characteristic changes with time, differences in the mRNA abundance were limited to lipogenesis-related genes on d −49 (HBCS-PH > HBCS-PN). The HBCS-PH had more than two-fold higher muscle concentrations of short (C2, C4-OH, C6-OH) and long-chain acylcarnitines (C16, C18, and C18:1) than HBCS-PN, indicating a greater oxidative capacity for fatty acids (and utilization of ketones) in muscle of HBCS-PN than HBCS-PH cows.

## Introduction

In dairy cows, insufficient nutrient intake during the first weeks of lactation coincides with a rapid increase of milk production and commonly results in a negative nutrient balance. Adipose tissue (AT), as the major energy reserve in mammals, plays a crucial role in the successful establishment and support of lactation^1^. During the periparturient period, fatty acids (FA) from AT are released into the circulation and are oxidized by hepatic and extrahepatic tissues as an energy source^2^, and thus AT is driven towards a catabolic state^3^. The rate of FA mobilization is under tight hormonal and neural control^4–6^. Growth hormone (GH) is known to stimulate lipolysis by enhancing the response and sensitivity of AT to the lipolytic stimulus of catecholamines^5–7^. Insulin acts as an antagonist stimulating lipid synthesis and inhibiting lipolysis^8^, and thus exerts a negative feedback on lipolysis, but peripheral insulin sensitivity and responsiveness, as well as reduced pancreatic insulin secretion are common during the periparturient period; as recently reviewed^9^. This contributes also to the uncoupling of the somatotropic axis shifting its actions away from the IGF-mediated anabolic effects to the direct lipid-catabolic effects of GH. Besides being a storage organ, AT is considered as an active endocrine organ that secretes bioactive factors known as adipocytokines or adipokines [e.g., leptin, adiponectin, and tumor necrosis factor α (TNFα)]. Adipokines act both locally and systemically and play a vital role in energy metabolism, regulation of feed intake and body weight, insulin sensitivity as well as in immune response^10,11^.

Using different machine learning (ML) algorithms on serum metabolomics data, we identified divergent metabotypes within a group of cows previously classified as over-conditioned based on body condition score (BCS) and backfat thickness (BFT): the high BCS (HBCS) group that was thereby predicted high (HBCS-PH; BCS > 3.75 and BFT > 1.4 cm) had metabolic profiles distinct from normal conditioned cows (NBCS). In contrast, the HBCS cows that were predicted as normal (HBCS-PN) had metabolic profiles alike NBCS cows (NBCS-PN; BCS < 3.5 and BFT < 1.2 cm; Ghaffari et al.)^12^. The HBCS-PN cows had greater feed and energy intakes but equal milk yields as the HBCS-PH^12^. Albeit this may point to decreased efficiency of milk production in early lactation, the greater intakes in HBCS-PN are likely beneficial for animal health at that time and thus may pay off by reducing losses due to diseases and allowing for efficient production in later stages of lactation. The circulating FA concentrations depend on their release from AT and their use for oxidation or lipogenesis. The increase of FA mobilization in periparturient cows arises from (1) promotion of lipolysis, (2) reduced intracellular re-esterification of FA released by lipolysis, (3) suppression of de novo synthesis or uptake of FA, and subsequent lipogenesis, and (4) a combination of all three^13^. The concentrations of FA in serum were not different between the different metabotypes of HBCS cows thus making differences in the aforementioned mechanisms likely. We therefore aimed at comparing the processes in subcutaneous AT (SCAT) in HBCS-PN and HBCS-PH cows and studied the mRNA abundance of genes associated with lipolysis and lipogenesis together with the expression of lipid-derived mediators. We hypothesized that the expression of these genes would differ between HBCS-PN and HBCS-PH despite a similar serum FA profile. We also assessed the skeletal muscle metabolome for testing whether there are indications for differences in the oxidative capacity of skeletal muscle in different metabotypes of over-conditioned periparturient cows.

## Results

### FA and β-hydroxybutyrate (BHB) concentrations in blood and mRNA expression results in SCAT

The concentrations of FA and BHB in serum (modified from Ghaffari et al.)^12^ are presented in the Supplemental Figure S1. In all groups, the serum concentrations of FA increased after calving (*P* < 0.01), and had greater concentrations in HBCS-PH and HBCS-PN than NBCS-PN in week 2, but without differences between HBCS-PN and HBCS-PH. In contrast, the serum concentrations of BHB were greater (*P* < 0.01) in HBCS-PH than in HBCS-PN and NBCS-PN during weeks 2–7 postpartum.

**Figure 1:**
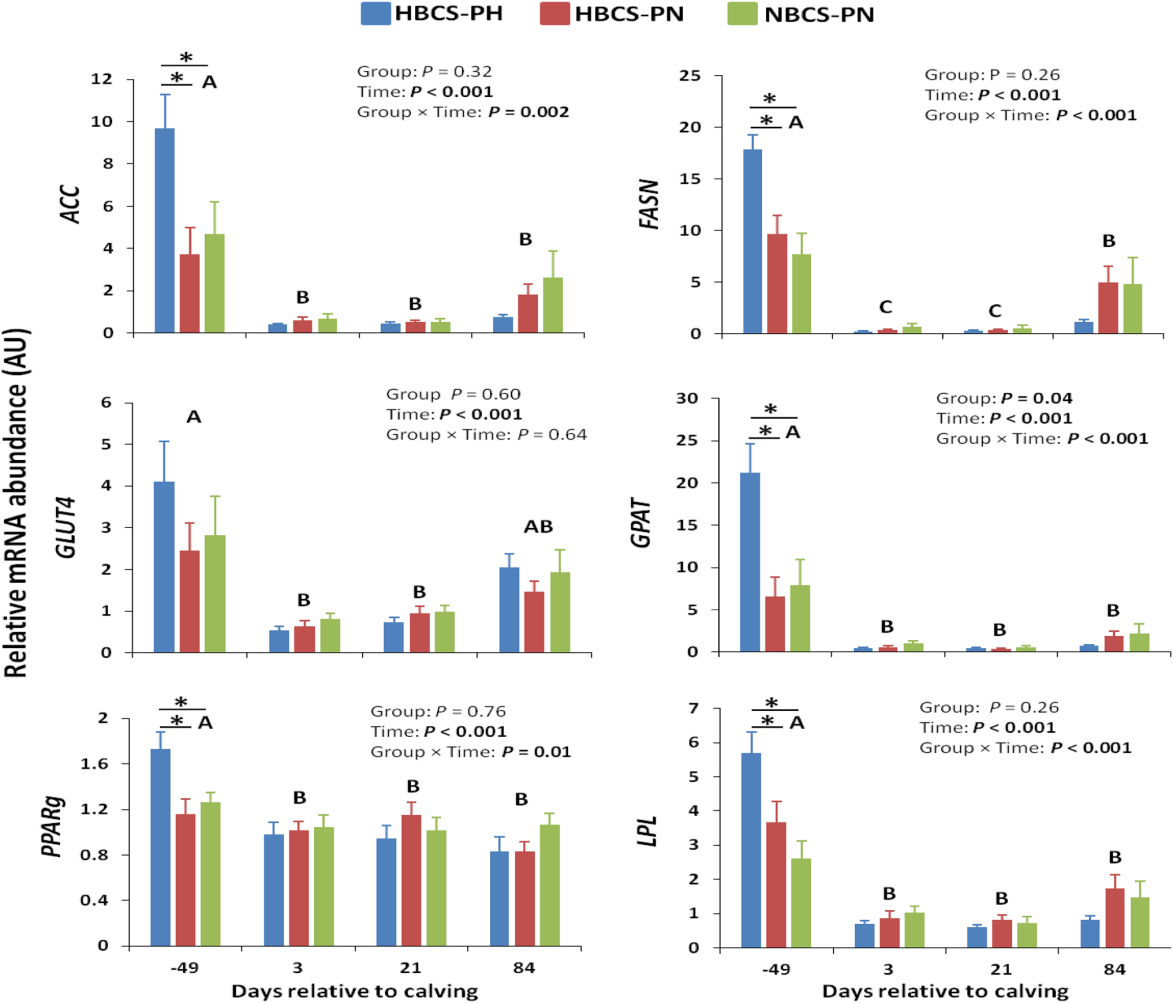
Time course of subcutaneous adipose tissue mRNA abundance (means ± SEM) of genes related to lipogenesis in high-body condition score (BCS) cows predicted high (HBCS-PH), high-BCS cows predicted normal (HBCS-PN), and normal-BCS cows predicted normal (NBCS-PN) during the observation period. Different letters (A, B) indicate differences (*P* < 0.05) between different time points (regardless of treatment). Asteristics indicate a significant difference (*P* < 0.05) between the groups within a given time point. *ACC* acetyl-CoA carboxylase, *FASN* fatty acid synthase, *GLUT4* glucose transporter 4, *GPAT* glycerol-3-phosphate acyltransferase, *PPARg* peroxisome proliferator-activated receptor γ, *LPL* lipoprotein lipase.

The mRNA abundance of key genes related to lipogenesis in SCAT is shown in Fig. 1. The mRNA abundance of acetyl-CoA carboxylase (*ACC*), fatty acid synthase (*FASN*), glycerol-3-phosphate acyltransferase (*GPAT*), peroxisome proliferator-activated receptor γ (*PPARg*), and lipoprotein lipase (*LPL*) on d −49 were greater (*P* ≤ 0.001) in HBCS-PH than in HBCS-PN and NBCS-PN. The abundance of glucose transporter 4 (*GLUT4*) mRNA did not differ between the groups. The mRNA abundance of all lipogenic genes changed over time (*P* < 0.001) and followed a similar pattern in all groups, having greater abundance prepartum than post partum.

**Figure 2:**
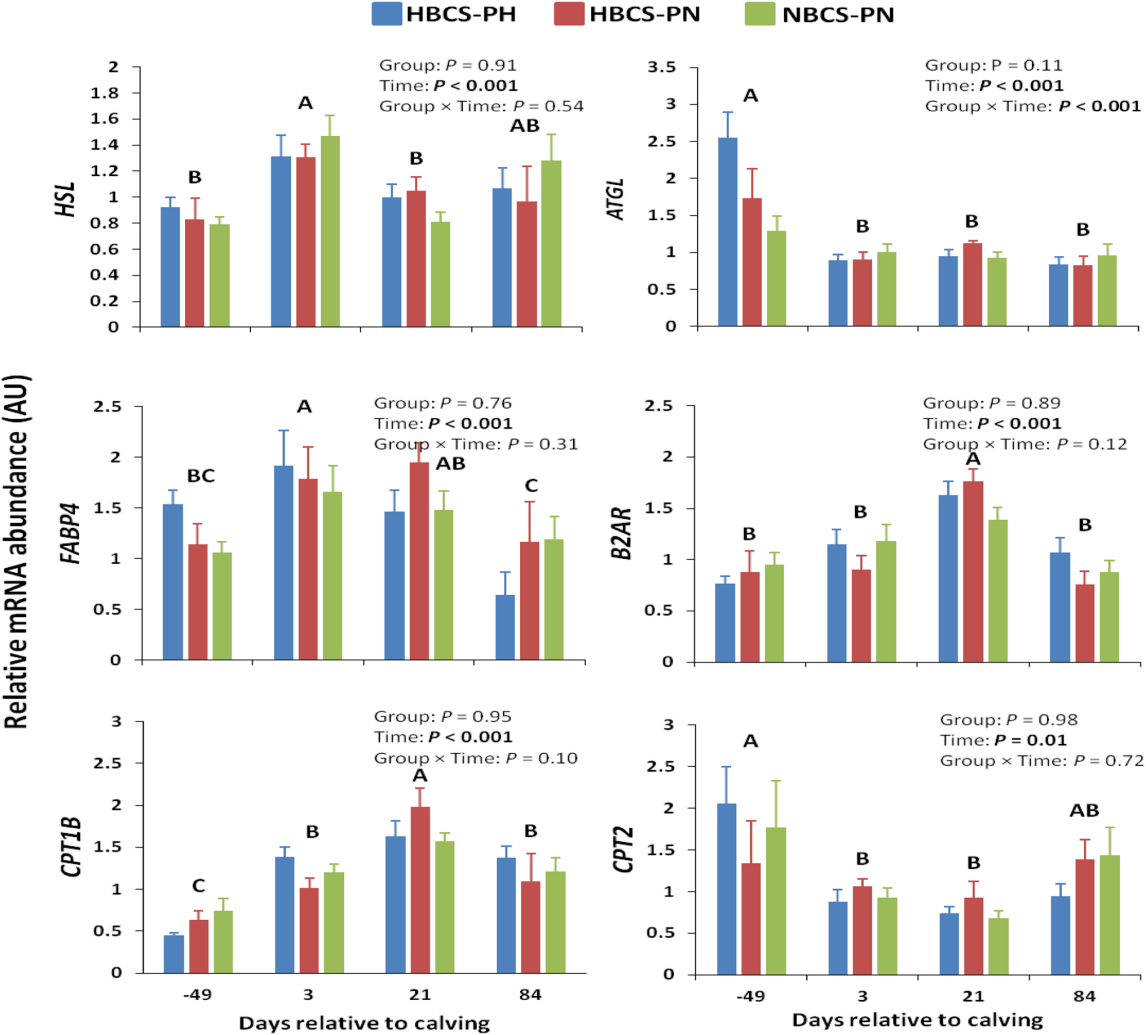
Time course of subcutaneous adipose tissue mRNA abundance (means ± SEM) of genes related to lipolysis and carnitine acyltransferases in high-body condition score (BCS) cows predicted high (HBCS-PH), high-BCS cows predicted normal (HBCS-PN), and normal-BCS cows predicted normal (NBCS-PN) during the observation period. Different letters (A–C) indicate differences (*P* < 0.05) between different time points (regardless of treatment). *HSL* hormone-sensitive lipase, *ATGL* adipose triglyceride lipase, *FABP4* fatty acid-binding protein 4, *β2AR* beta-2 adrenergic receptor, *CPT1B* carnitine palmitoyltransferase 1B, *CPT2* carnitine palmitoyltransferase 2.

The mRNA abundance of key genes related to lipolysis and of the carnitine acyltransferases in SCAT is shown in Fig. 2. The mRNA abundance of hormone-sensitive lipase (*HSL*), adipose triglyceride lipase (*ATGL*), fatty acid-binding protein 4 (*FABP4*), beta-2 adrenergic receptor (*β2AR*), carnitine palmitoyltransferase (*CPT*)*1B*, and *CPT2* did not differ among groups, but changed over time and followed almost a similar pattern in all groups (except for *ATGL* and *CPT2*); that is, they increased from d −49 to d 3 and 21 and decreased thereafter.

**Figure 3:**
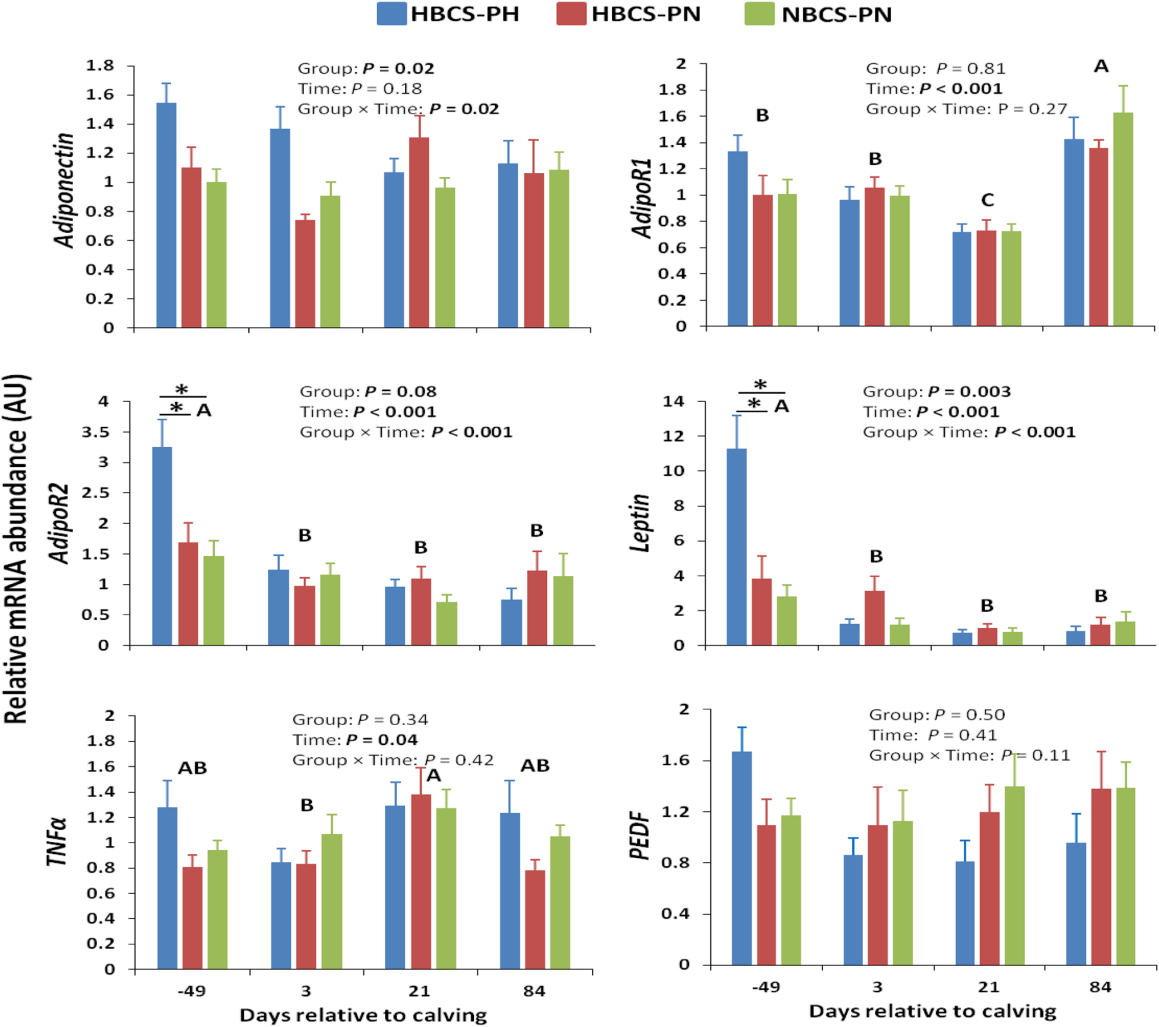
Time course of subcutaneous adipose tissue mRNA abundance (means ± SEM) of fat-derived bioactive factors in high-body condition score (BCS) cows predicted high (HBCS-PH), high-BCS cows predicted normal (HBCS-PN), and normal-BCS cows predicted normal (NBCS-PN) during the observation period. Different letters (A–C) indicate differences (*P* < 0.05) between different time points (regardless of treatment). Asteristics indicate a significant difference (*P* < 0.05) between the groups within a given time point. *AdipoR1* adiponectin receptor 1, *AdipoR2* adiponectin receptor 2, *TNFα* tumor necrosis factor α, *PEDF* pigment epithelium-derived factor.

The mRNA abundance of some adipokines is presented in Fig. 3. The differences observed in the mRNA abundance of these factors among groups were limited to *leptin* and adiponectin receptor 2 (*AdipoR2*) on d −49; that is, they were greater (*P* ≤ 0.001) in HBCS-PH than in HBCS-PN and NBCS-PN. The mRNA abundance of *TNFα* and adiponectin receptor 1 (*AdipoR1*) changed over time (*P* ≤ 0.04), but that of pigment epithelium-derived factor (*PEDF*) was not affected by time and there was no group × time interaction.

### Muscle metabolome profiles

**Figure 4:**
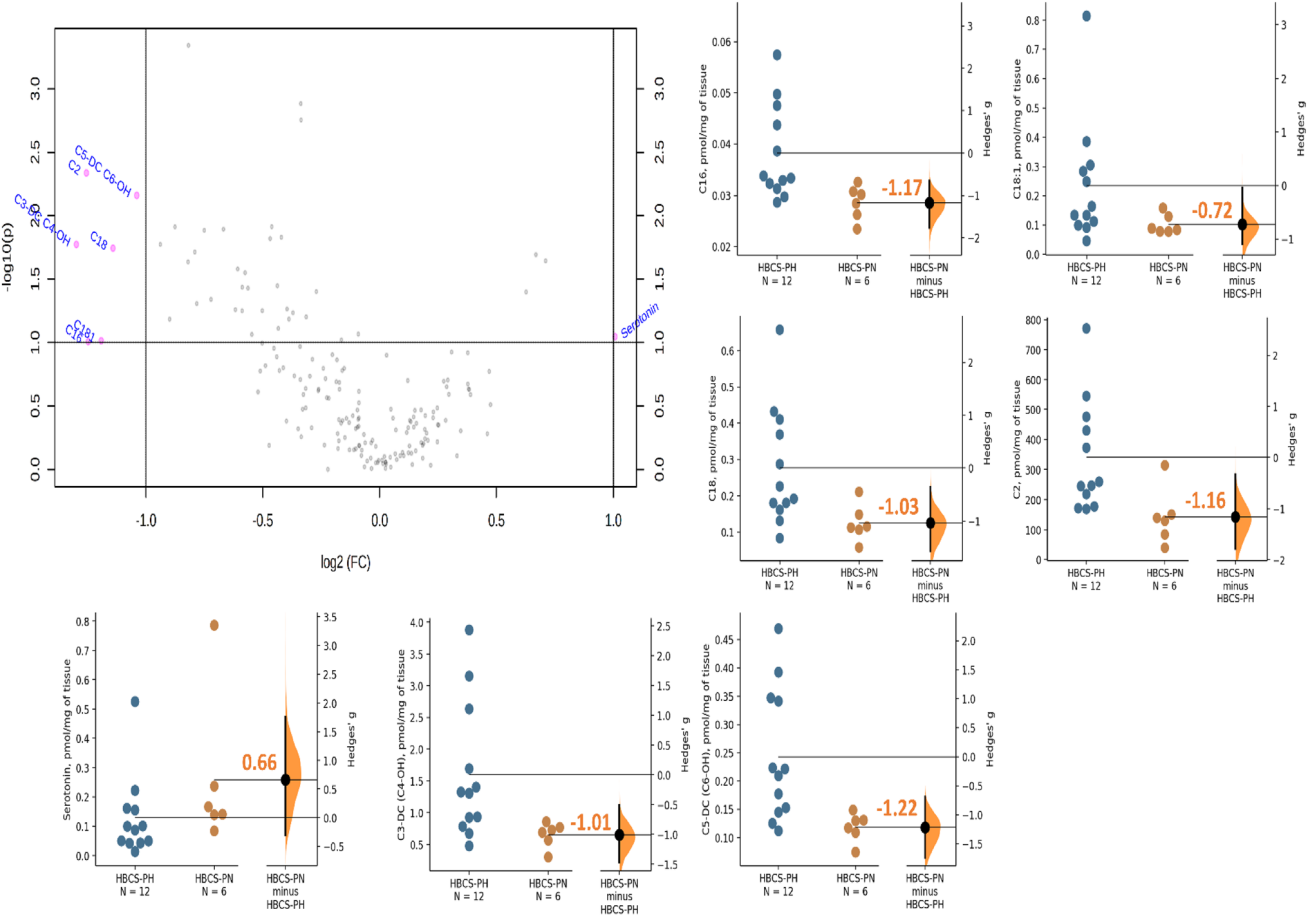
Volcano plot visualizing muscle metabolites that differ between high-body condition score (BCS) cows predicted high (HBCS-PH) and high-BCS cows predicted normal (HBCS-PN) on d 21 relative to calving. The x-axis represents the mean of log_2_ fold-change value, and the y-axis corresponds to the negative logarithm of the *P*-values. Each circle represents a single metabolite. The dashed horizontal line represents the level of significance for the t-tests performed (0.10), and the vertical dashed lines display the threshold set for fold-change (2). The red circles show significantly changed metabolites; the gray circles designate metabolites that were not changed. Effect size, shown as Hedges' g between HBCS-PN and HBCS-PH is shown in the above Gardner-Altman estimation plot of muscle acetylcarnitine (C2), hydroxybutyrylcarnitine (C4-OH (C3-DC)), hydroxyhexanoylcarnitine (C6-OH (C5-DC)), hexadecanoylcarnitine (C16), octadecanoylcarnitine (C18), octadecenoylcarnitine (C18:1), and serotonin.

The univariate analysis with volcano plot was performed to obtain an overview of the metabolites that discriminate most significantly the different metabotypes of over-conditioned dairy cows (HBCS-PN vs. HBCS-PH) on d 21 (Fig. 4). The muscle concentrations of acetylcarnitine (C2; effect size, *g* = − 1.16), hydroxybutyrylcarnitine (C4-OH; *g* = − 1.01), hydroxyhexanoylcarnitine (C6-OH; *g* = − 1.22), hexadecanoylcarnitine (C16; *g* = − 1.17, octadecanoylcarnitine (C18; *g* = − 1.03), and octadecenoylcarnitine (C18:1; *g* = − 0.72) were lower in HBCS-PN than in HBCS-PH, whereas those of serotonin (*g* = 0.69) were greater.

## Discussion

In the current study, we hypothesized that expression of key factors known to regulate lipolysis and lipogenesis in SCAT differs between HBCS-PN and HBCS-PH despite a similar serum FA profile. In contrast to our hypothesis, the changes observed in the abundance of genes associated with lipolysis and lipogenesis in the SCAT of different metabotypes in over-conditioned dairy cows were limited to d −49 and the observed differences largely disappeared thereafter. We observed greater mRNA abundance of *LPL*, *ACC*, *FASN*, *GPAT*, and *PPARg* in HBCS-PH compared with HBCS-PN and NBCS-PN on d −49. In AT, FA originate from both uptake from the circulation and from de novo synthesis^4^. Fatty acid uptake is facilitated by the extracellular activity of LPL. Lipoprotein lipase activity is regulated by nutritional and endocrine status^4,23^. Acetyl-CoA carboxylase and FASN are considered as key enzymes involved in the de novo synthesis of FA. Moreover, GPAT catalyzes the first step of the synthesis of triacylglycerol^24,25^. It has been shown that *PPARg* gene expression in dairy cattle is linked to lipogenic gene expression in the mammary gland and that PPARg plays an important role in adipocyte differentiation and improves insulin sensitivity through increasing glucose uptake and by the induction of genes related to FA metabolism and FA uptake^26^. Thus, these changes would result in increased lipogenesis in HBCS-PH as reflected by greater BCS and BFT in these cows compared to the HBCS-PN cows on d −49; though, both over-conditioned groups had identical body condition around calving. These results show that different metabotypes of over-conditioned cows responded to the same diet, received from weeks 15 to 7 before calving, differently. We observed a lower abundance of lipogenic gene mRNA abundance on d 3, 21, and 84 compared with d −49 in all groups. Therefore, potentially lower availability of lipoprotein-derived FA (due to decreased LPL), de novo synthesis of FA (due to decreased ACC and FASN) and lower intracellular (re-)esterification of FA to triacylglycerol (due to decreased GLUT4 and GPAT) may occur in SCAT in early lactation compared with prepartum. Lower mRNA abundance^27,28^, as well as protein activity^29^ of the key factors known to regulate lipogenesis in AT, have already been reported in postpartum dairy cows. In AT, insulin promotes glucose transport and lipogenesis, whereas energy deficit regulates them negatively^6,30,31^. This means that the physiological state associated with the initiation of galactopoiesis in dairy cows favors the downregulation of lipogenic genes in SCAT and, thus, energy partitioning towards the mammary gland independent of body condition.

In the dairy cow, early lactation is associated with low insulin and high GH concentrations coinciding with adrenergic signaling, which favors mobilization of body fat depots, representing a homeorhetic adaptation to ensure that the mammary gland is adequately supplied with nutrients^4,6,23^. Insulin, the main antagonist of adrenergic signaling, is an anti-lipolytic hormone; insulin resistance thus can lead to further mobilization of lipid from AT in dairy cows. Several adipokines such as TNFα, adiponectin, leptin, and PEDF have been implicated as potential mediators of insulin resistance, both local and systemic^32–34^. In the current study, the mRNA abundance of key factors known to regulate lipolysis and insulin sensitivity in the SCAT did not differ between different metabotypes of over-conditioned dairy cows post partum. This may suggest lipolytic response and likely insulin sensitivity of comparable magnitude in the SCAT of these cows. However, it should be noted that our evaluation in the current study was conducted using tail head SCAT, and findings from the present study thus apply to this depot, and may not be representative for other body fat depots, in particular visceral fat.

As shown in the companion study by Ghaffari et al., the serum concentrations of leptin, adiponectin, glucose, and insulin were similar between HBCS-PN and HBCS-PH across all time-points^12^. We thus propose two possible scenarios for the difference in the BHB but not the FA serum concentrations of HBCS-PH and HBCS-PN cows. First, that the oxidative capacity for FA in other tissues, such as skeletal muscle, contributed more to reducing the metabolic load of FA on the liver in HBCS-PN than in HBCS-PH. This scenario is supported by more than twofold higher muscle concentrations of C2, C4-OH (C3-DC), C6-OH (C5-DC), and long-chain acylcarnitines (C16, C18, and C18:1) on d 21 in HBCS-PH than in HBCS-PN. Although the majority of acylcarnitines is derived from FA oxidation, other intermediates including ketone bodies (C4-OH), degradation products of lysine, tryptophan, valine, leucine, and isoleucine (C3 and C5), and carbon atoms from glucose (C2) also yield acylcarnitines. To generate energy from long-chain FA, they are transported across the inner mitochondrial membrane from the cytoplasm into mitochondria by a carnitine-dependent transport shuttle^35,36^. Inside the mitochondria, carnitine and long-chain acyl-CoA are regenerated that then can enter the β-oxidation pathway followed by complete oxidation of the resultant acetyl-CoA in the tricarboxylic acid (TCA) cycle and respiratory chain^37^. However, impaired function of the carnitine shuttle system or depletion of TCA cycle intermediates leads to incomplete mitochondrial FA oxidation, resulting in accumulation of acylcarnitines and also oxidative stress, which are parts of the metabolic events leading to insulin resistance in skeletal muscle, as documented in human studies^38–40^. In this situation, the accumulated (long-chain) acylcarnitines in the mitochondrial matrix are subsequently transported out of the mitochondria to the bloodstream^35,37^. In support of this, in a companion study, Ghaffari et al. found greater serum concentrations of C2, propionyl-carnitine (C3), butyryl-carnitine (C4), and long-chain acylcarnitines (C16, C18, and C18:1) in HBCS-PH than in HBCS-PN on d 21^12^. However, the potential contribution of the liver with a large capacity for mitochondrial FA oxidation to acylcarnitine turnover is also very likely, but needs to be tested in dairy cows.

Another plausible but speculative scenario for these results is that the contribution of visceral AT (whose venous drainage is via the portal vein) lipolysis to hepatic FA delivery increased to a greater extent in HBCS-PH than in HBCS-PN. Consequently, as lipolysis increases, the liver would be directly “flooded” with FA from visceral AT mobilization, which might not be detected by the changes in the systemic circulation. The FA overload is primarily handled by the liver through increasing FA oxidation. However, when the rates of FA β-oxidation exceed those of downstream metabolic pathways, such as the TCA cycle and respiratory chain, this will lead to a program of hepatic ketogenesis and, most likely, efflux of acylcarnitines into the circulation. This speculation is supported by greater serum BHB as well as short- (C2, C3, and C4) and long-chain (C16, C18, and C18:1) acylcarnitines^12^ in HBCS-PH than in HBCS-PN post partum. The physiological reason for the efflux of acylcarnitines to the bloodstream is not clear, but it seems that acylcarnitine formation prevents CoA trapping, allowing preservation of a sufficient pool of CoA in the matrix for CoA-dependent metabolic processes^41,42^. Besides plasma, acylcarnitines are also detectable in bile and urine^43,44^, proposing that acylcarnitines excretion may also serve as a detoxification process. Taken both scenarios together, greater muscle and serum acylcarnitines in HBCS-PH than in HBCS-PN reflect impaired metabolic flexibility in the former cows; HBCS-PN likely are more capable of metabolizing FA and utilize BHB as fuel (reflected by lower C4-OH in muscle).

## Conclusion

Contrasting our hypothesis, the mRNA abundance of genes associated with lipolysis and lipogenesis in SCAT of different metabotypes identified within a cohort of over-conditioned dairy cows differed only on d −49 and the observed differences largely disappeared thereafter. The higher abundance of *LPL*, *ACC*, *FASN*, *GPAT*, and *PPARg* mRNA on d −49 in HBCS-PH than in HBCS-PN may suggest an increased lipogenesis in HBCS-PH. Lack of changes in the mRNA abundance of key factors known to regulate lipolysis and insulin sensitivity in the SCAT might reflect lipolytic response and likely insulin sensitivity of comparable magnitude in the SCAT of different metabotypes of over-conditioned dairy cows. Differences in the oxidative capacity for FA (and utilization of ketone bodies) in tissues other than liver, such as skeletal muscle may contribute to the differences in BHB, but not FA serum concentrations between HBCS-PH and HBCS-PN. The data show that tissue and circulating acylcarnitines profiles reflect important traits of intermediary metabolism that contribute to understanding the metabolic stages in over-conditioned cows.

## Materials and methods

### Basic trial: comparison of high versus normal body condition

The experiment was conducted at the Educational and Research Centre for Animal Husbandry, Hofgut Neumuehle, Muenchweiler a.d. Alsenz, Germany. The experimental procedures performed in this study were in accordance with the European Union Guidelines concerning the protection of experimental animals, with approval by the local authority for animal welfare affairs (Landesuntersuchungsamt Rheinland-Pfalz, Koblenz, Germany [G 14-20-071]). The study is reported according to the ARRIVE guidelines. The animals were part of a trial aiming to establish an experimental model of high versus normal body reserve mobilization around calving. Details of the experimental design, performance data, as well as classical metabolites and metabolic hormones have been described previously^14^. In brief, fifteen weeks before calving, 38 multiparous German Holstein cows (average parity 2.9 ± 0.3; mean ± SEM) were allocated to either a normal-conditioned (NBCS; n = 19) or high-conditioned group (HBCS; n = 19). In order to reach the targeted differences in BCS (a 5-point scale: 1 = emaciated and 5 = extremely fat with 0.25 increments)^15^ and backfat thickness (BFT) in the experimental groups (NBCS: BCS < 3.5 and BFT < 1.2 cm; HBCS: BCS > 3.75 and BFT > 1.4 cm) until dry-off (week 7 ante partum), NBCS cows received a less energy-dense ration (6.8 MJ NE_L_/kg DM) than the HBCS cows (7.2 MJ NE_L_/kg DM) during late lactation (from week 15 to 7 before the anticipated calving date). During the dry period and subsequent lactation, both groups received identical diets. The diets were fed as total mixed ration (TMR) for ad-libitum intake consisting of 63% roughage and 37% concentrate in the high-energy diet, or 74% roughage, and 26% concentrate in the low-energy diet. The diets were formulated to meet the nutritional requirements of dairy cows according to the recommendation of the Society of Nutrition Physiology^16^.

### Current trial: metabolic clustering of dairy cows

The metabolic clustering of the animals using different ML algorithms and serum metabolomics data has been described in the companion study by Ghaffari et al.^12^. Metabolic clustering by applying four supervised ML-based classifiers (sequential minimal optimization, random forest, alternating decision tree, and naïve Bayes–Updatable) on the delta changes in concentrations of 170 serum metabolites [day 21 post partum minus d 49 ante partum; Targeted metabolomic analyses using the AbsoluteIDQ™ p180 Kit (Biocrates Life Sciences AG, Innsbruck, Austria)] resulted in 4 distinct metabolic clusters of HBCS predicted HBCS (HBCS-PH, n = 13), HBCS predicted NBCS (HBCS-PN, n = 6), NBCS predicted NBCS (NBCS-PN, n = 15), and NBCS predicted HBCS (NBCS-PH, n = 4). Due to the low number of NBCS-PH cows, we did not consider this group for further comparisons.

### Blood sampling and analysis

Blood samples were collected weekly (from week 7 ap until week 12 pp) from the tail vein after the morning milking before cows had access to the new fresh ration and before collecting SCAT biopsies for analyzing FA and BHB as previously described^12,14^.

### Adipose tissue biopsies

SCAT samples were collected from the tail head region with a scalpel through an incision of 1 cm width on −49, 3, 21, and 84 relative to calving under local anesthesia (procaine hydrochloride, 20 mg/mL, 9 mL per biopsy; Richter Pharma AG, Wels, Austria) while the animals were sedated (xylazine i.v., 20 mg/mL, 0.1 mL/100 kg BW; CP-Pharma Handels GmbH, Burgdorf, Germany) and fixed in a headlock. Immediately after sampling, incisions were closed with a sterile needle and sterile absorbable suture (Spool suture PGA, USP 1, EP 4, LOT 15B27, Henry Schein U.K. Holdings Ltd, Gillingham, UK). To prevent infection and for analgesia, respectively, oxytetracycline hydrochloride was applied to the skin (25 mg/mL, EngemycinTM, MSD Animal Health Innovation GmbH, Schwabenheim an der Selz, Germany) and a ketoprofen injection (100 mg/mL, 3 mL/100 kg BW; Streuli Pharma AG, Uznach, Switzerland) was given. Tissue samples were rinsed with 0.9% NaCl to remove any blood contamination, immediately snap-frozen in liquid nitrogen, and stored at − 80 °C until time of analysis.

### RNA extraction and quantitative real-time reverse transcription-PCR

Detailed protocols for the preparation of the AT samples including RNA extraction and cDNA synthesis were described previously^17^. The integrity of the RNA was checked using ethidium bromide denaturing RNA gel electrophoresis. Only samples with clear 28S and 18S rRNA bands at an approximate intensity ratio of 2:1 and an A260/280 absorbance ratio of 1.8–2.1 were used for cDNA synthesis and subsequent RT-qPCR. The sequences of the primer pairs as well as the real-time PCR conditions are provided in Table 1. Analysis by quantitative real-time RT-PCR was carried out in an Mx3000P cycler (Agilent, Santa Clara, CA) and in accordance with the Minimum Information for Publication of Quantitative Real-Time PCR Experiments (MIQE) guidelines^18^.

**Table 1:** Characteristics of primers and real-time polymerase chain reaction conditions.

Gene	Sequences (5'–3')	NCBI accession no.^a^	Length (bp)	Annealing (s/◦C)^b^
***HSL***				
Forward	CTTCTTTGAGGGTGATGAG	NM_001080220.1	180	60/60
Reverse	GTCTCGTTTCGTTTGTAGTG			
***β2AR***				
Forward	TCCGCTTTCAATCCCCTTATC	Z86037	156	60/59
Reverse	TCCACTCTGTTCCCCTGTGTAG			
***PPARg***				
Forward	ATTGGTGCGTTCCCAAGTTT	Y12420.1	57	60/60
Reverse	GGCCAGTTCCGTTCAAAGAA			
***CPT1B***				
Forward	GCAGATGATGGCTATGGA	NM_001034349.2	78	20/61
Reverse	GGAGAACTTGCTGGAGAC			
***CPT2***				
Forward	GTAGCCAGTAAGCACTATTC	NM_001045889.2	180	60/59
Reverse	CCAAGTCTTACCTCCTGATA			
***Leptin***				
Forward	GACATCTCACACACGCAG	U62123	183	30/60
Reverse	GAGGTTCTCCAGGTCATT			
***TNFα***				
Forward	TGCCTGCTGCACTTCGGGGTA	EU276079.1	50	60/60
Reverse	CCTGGGGACTCTTCCCTCTGGGG			
***PEDF***				
Forward	GGACTGGAGCCCTGCTTGGGT	AF017058	79	60/60
Reverse	CCGTGCTCTCAGGGGTCAGA			
***ATGL***				
Forward	CGTGTCTCTGATGGCGAGAA	NM_001046005.2	71	30/60
Reverse	ACATTGGCCTGGATAAGCTCC			
***GLUT4***				
Forward	ACCTTATGGCCACTCCTCCT	NM_174604.1	180	65/60
Reverse	CTCAGCCAACACCTCAGACA			
***GPAT***				
Forward	CAGATGAATCCCGCCGAAGA	NM_001012282	133	30/61
Reverse	CCAATTCCCTGCCTGTGTCT			
***LPL***				
Forward	AACCGGCTTAGATCCAGCTG	NM_001075120	251	30/60
Reverse	GCTGATCCACATCTCCAAGG			
***ACC***				
Forward	CGTTTGGGGTTATTTCAGTG	NM_174224	105	60/60
Reverse	ATTGCTTCCTCTCGGTTTTC			
***FABP4***				
Forward	CATCTTGCTGAAAGCTGCAC	X89244	160	30/60
Reverse	AGCCACTTTCCTGGTAGCAA			
***Adiponectin***				
Forward	CCTGACTGAAGTCTGTGGCTC	NM_174742.2	113	60/60
Reverse	CTTCCATGTTGTCCTCGCCA			
***AdipoR1***				
Forward	GCTGAAGTGAGAGGAAGAGTC	NM_001034055	118	45/60
Reverse	GAGGGAATGGAGTTTATTGCC			
***AdipoR2***				
Forward	GGCAACATCTGGACACATC	NM_001040499.2	200	45/60
Reverse	CTGGAGACCCCTTCTGAG			
***FASN***				
Forward	ACCTCGTGAAGGCTGTGACTCA	NM_001012669.1	92	30/60
Reverse	TGAGTCGAGGCCAAGGTCTGAA			

*HSL* hormone-sensitive lipase, *β2AR* β2-adrenergic receptor, *PPARg* peroxisome proliferator activated receptor gamma, *CPT1B* carnitine palmitoyltransferase 1B, *CPT2* carnitine palmitoyltransferase 2, *TNFα* tumor necrosis factor α, *PEDF* pigment epithelium-derived factor, *ATGL* adipose triglyceride lipase, *GLUT4* glucose transporter type 4, *GPAT* glycerol-3-phosphate acyltransferase, *LPL* lipoprotein lipase, *ACC* acetyl-CoA carboxylase, *FABP4* fatty acid-binding protein 4, *AdipoR1* adiponectin receptor 1, *AdipoR2* adiponectin receptor 2, *FASN* fatty acid synthase.

^a^National Center for Biotechnology Information (NCBI) accession number.

^b^Initial denaturation for 10 min at 90 °C; denaturation for 30 s at 95 °C; Extension for 30 s at 72 °C.

The RT-PCR amplifications were set up in a total volume of 10 µL consisting of 2 µL cDNA (diluted 1:4) as a template, 1 µL assay-specific primer mix, 2 µL of water, and 5 µL of the DyNAmo ColorFlash SYBR Green qPCR Kit (Thermo Scientific, Dreireich, Germany). Each run included a negative-template control for quantitative PCR, as well as a negative-template control and no-reverse transcriptase control of cDNA. For each PCR, a standard curve was generated using a serial-diluted cDNA as the template to calculate efficiency-corrected relative quantities of the targets (run-specific target amplification efficiency). A set of two inter-run calibrators was included for each PCR plate. The mRNA abundance of the target genes was normalized with the four most stable reference genes, namely hippocalcin-like 1 (*HPCAL1*), emerin (*EMD*), RNA polymerase II (*POLR2A*), and eukaryotic translation initiation factor 3 (*EIF3K*), which were evaluated based on their average expression stability (M) and pairwise variation (V) values (M < 1.0 and V = 0.15) using the geNorm^PLUS^ algorithms of qBase^PLUS^ 3.2 software (Biogazelle, Ghent, Belgium). The output data from the software were calibrated normalized relative quantities that were used for statistical analysis of the mRNA data.

### Profiling of the skeletal muscle metabolome

The metabolome was characterized in muscle samples (*M. semitendinosus*) obtained on d 21 using a targeted quantitative metabolomics approach by a commercially available kit (AbsoluteIDQ p180 kit; Biocrates Life Sciences AG, Innsbruck, Austria) as previously described for comparing the HBCS with the NBCS group^19^. The metabolite panel consists of 188 different metabolites, including 21 amino acids, 40 acylcarnitines, 15 sphingomyelins, 90 glycerophospholipids, 21 biogenic amines, and hexoses (H1; the sum of hexoses – about 90–95% glucose).

### Statistical analyses

Statistical analyses of the mRNA data, as well as of serum FA and BHB, were performed using repeated measures in the MIXED procedure of SAS software (version 9.3; SAS Institute Inc., Cary, NC). The model consisted of treatment, time (day relative to calving), and interaction of treatment and time as fixed effects, and cow as the random effect. The appropriate covariance structure for analyzing repeated measures data was specified using the Akaike information criterion and Bayesian information criterion. The Tukey–Kramer adjustment was applied to control the effect of multiple comparisons. Statistical significance was declared at *P* ≤ 0.05, and a trend to significance was declared at 0.05 < *P* ≤ 0.10.

Muscle metabolome data were analyzed with MetaboAnalyst 4.0^20^. Data were processed by log transformation and Pareto scaling before the statistical analysis. Univariate analysis including fold-change analysis and volcano plots were performed to assess the differences of detected metabolites between HBCS-PH and HBCS-PN groups and to obtain an overview and ranking of potentially important metabolites. Important metabolites were identified by volcano plot based on a fold-change threshold of 2 on the x-axis and t-tests threshold (*P*-value) of 0.1 on the y-axis. The muscle metabolites identified by volcano plot were further analyzed with estimation methods and presented as mean-difference estimation plots^21^. The effect size was measured using Hedges' g^22^ to assess the magnitude of the difference in muscle metabolome and classified as 0.2 having a smaller effect, 0.5 having a medium effect, and 0.8 having a larger effect size.

## Supplementary Information

Supplementary Figure S1.
