# Supplementary material for: Muscle metabolome and adipose tissue mRNA expression of lipid metabolism-related genes in over-conditioned dairy cows differing in serum-metabotype
Source: Sci Rep. 2021 May 27;11:11106. doi: 10.1038/s41598-021-90577-w (PMC8159933; doi:10.1038/s41598-021-90577-w)
Supplement: Supplementary file 1 — Supplementary Figure S1. [file 41598_2021_90577_MOESM1_ESM.pdf]

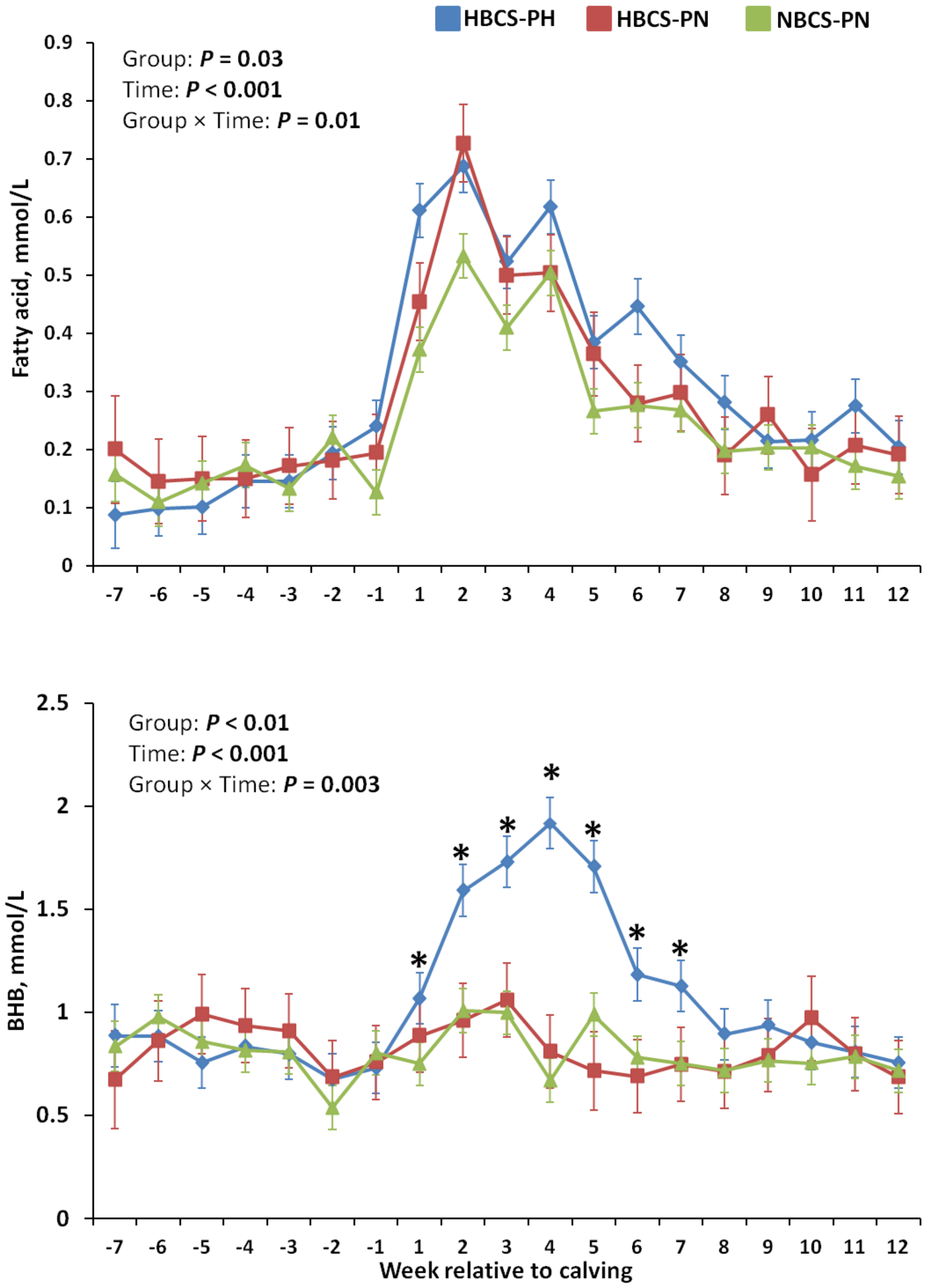

Supplemental Figure S1. Time course of the concentrations of fatty acids and β-hydroxybutyrate (BHB) of high-body condition score (BCS) cows predicted high (HBCS-PH), high-BCS cows predicted normal (HBCS-PN), and normal-BCS cows predicted normal (NBCS-PN) during the observation period (modified from Ghaffari *et al.*)[12]. Asterisks indicate differences ($P < 0.01$) between HBCS-PH vs. HBCS-PN and NBCS-PN at a given time point. Data are presented as mean ± SEM.
